# Rapid host expansion of an introduced parasite, the spiny rat louse *Polyplax spinulosa* (Psocodea: Phthiraptera: Polyplacidae), among endemic rodents in Australia

**DOI:** 10.1186/s13071-020-3957-y

**Published:** 2020-02-18

**Authors:** Wei Wang, Lance A. Durden, Renfu Shao

**Affiliations:** 1 GeneCology Research Centre, School of Science and Engineering, University of the Sunshine Coast, Maroochydore, QLD 4556 Australia; 2 Department of Biology, Georgia Southern University, Statesboro, GA 30458 USA

**Keywords:** Sucking lice, Rodents, Host-parasite relationships, Invasive species, Psocodea, Phthiraptera, Polyplacidae, *Polyplax spinulosa*

## Abstract

**Background:**

Historical European exploration and colonization resulted in the introduction of four species of rodents to the Australian continent from Eurasia: the brown rat, *Rattus norvegicus*, the black rat, *R. rattus*, the Pacific rat, *R. exulans*, and the house mouse, *Mus musculus*. The spread of these rodents created opportunities for their co-introduced sucking lice to parasitize and adapt to endemic rodents in Australia.

**Methods:**

We collected sucking lice from rodent specimens in seven museums across Australia. We identified the spiny rat louse, *Polyplax spinulosa*, based on morphology. We sequenced the mitochondrial *cox*1 and *rrnL* genes of *P. spinulosa* specimens and constructed a phylogenetic tree with *rrnL* sequences.

**Results:**

We examined 989 rodent specimens of 54 species and collected 2111 adult sucking lice and 1064 nymphal sucking lice. We found that *P. spinulosa* had nearly doubled its host range by parasitizing at least six endemic rodent species in Australia. The other two introduced lice, *P. serrata* and *Hoplopleura pacifica*, however, have apparently failed to expand to any endemic rodents in Australia. Our analysis of mitochondrial *rrnL* gene sequences divided *P. spinulosa* into two genotypes (European *vs* Southeast Asian), which differ by 7.5%; both genotypes were introduced into Australia and then expanded their host ranges to include endemic rodents.

**Conclusions:**

The earliest record of a European ship landing in Australia was in 1606, followed by British settlement in 1788. The expansion of *P. spinulosa* to at least six endemic rodent species in Australia has therefore occurred in the time frame of 200 to 400 years, which is extremely rapid relative to its host expansion to eight native rat species in Eurasia in ~ 16 million years since it diverged from *P. serrata*. The host expansion of *P. spinulosa* is remarkable for a blood-sucking louse and is in stark contrast to the absence of host expansion by *P. serrata* and *H. pacifica*. Comparison among these three introduced sucking lice indicated that both louse-specific factors and host-specific factors can contribute to the success or failure of host expansion.
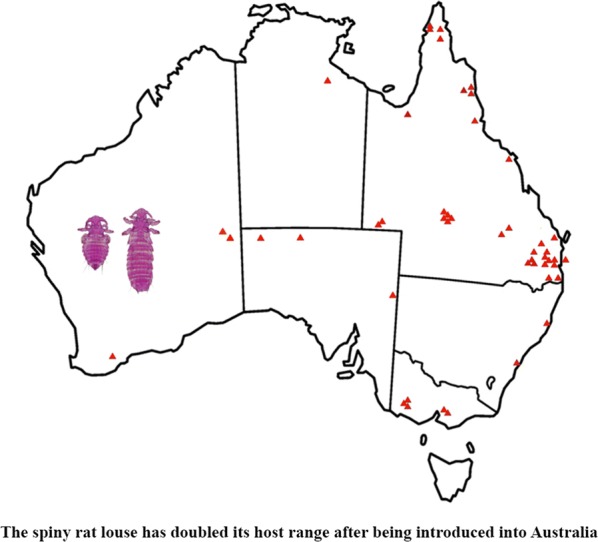

## Background

Blood-sucking lice (Psocodea: Phthiraptera: Anoplura) are wingless obligate permanent ectoparasites of eutherian mammals, with piercing mouthparts and dorso-ventrally flattened, almost oval-shaped bodies [1, 2]. Several species of sucking lice are known as disease vectors, transferring pathogens to hosts and causing louse-borne diseases [3–6]. Heavy infestation of sucking lice can also cause host hypersensitivity, dermatitis and even anemia [3, 6]. More than 540 species of sucking lice, which are all in the suborder Anoplura, have been described from 840 species of eutherian mammals [7]. Sucking lice are highly host-specific in comparison to other ectoparasites such as some chewing lice and most mites, ticks and fleas [3, 8]. Most sucking louse species parasitize a single host species or even a specific region of the host body, e.g. in humans, head lice are found only on head hair, body lice on clothes and pubic lice mainly on pubic hair [7, 9]. A small proportion of sucking louse species parasitize two or more host species [7], e.g. the spiny rat louse, *Polyplax spinulosa* (Burmeister, 1839) (Polyplacidae), has been recorded from nine species of rats: black rat, *Rattus rattus* (Linnaeus, 1758) (Asia), brown rat, *Rattus norvegicus* (Berkenhout, 1796) (Eurasia), greater bandicoot rat, *Bandicota indica* (Bechstein, 1800) (Asia), Asian rat, *Rattus tanezumi* (Temminck, 1844) (Asia), Himalayan field rat, *Rattus nitidus* (Hodgson, 1845) (Asia), Turkestan rat, *Rattus pyctoris* (Hodgson, 1845) (Asia), Polynesian rat, *Rattus exulans* (Peale, 1848) (Southeast Asia), long-haired rat, *Rattus villosissimus* (Waite, 1898) (Australia), and rice field rat, *Rattus argentiventer* (Robinson & Kloss, 1916) (Southeast Asia) [7, 10].

**Fig. 1:**
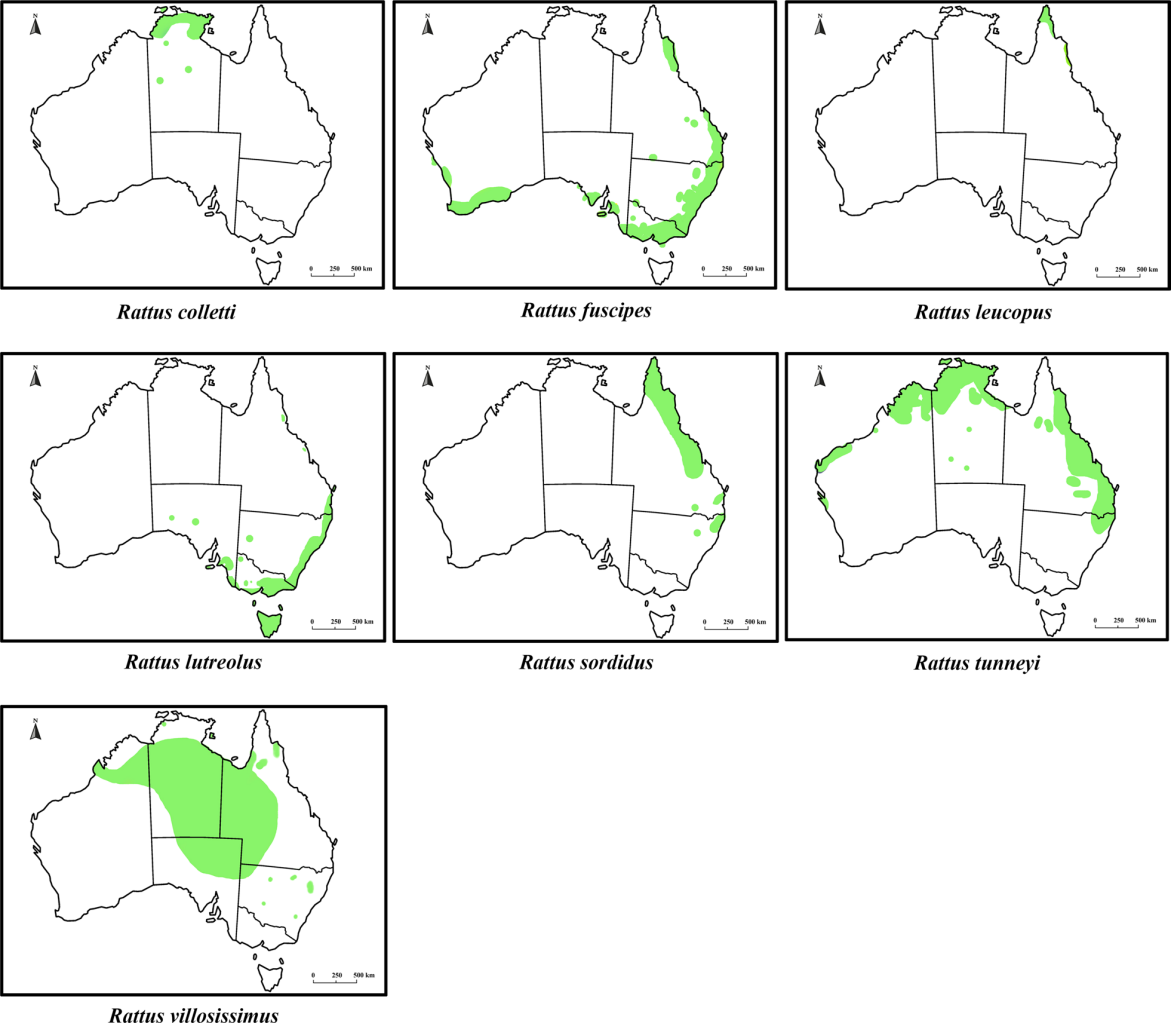
Geographical distribution of *Rattus colletti*, *Rattus fuscipes*, *Rattus leucopus*, *Rattus lutreolus*, *Rattus sordidus*, *Rattus tunneyi* and *Rattus villosissimus* in Australia (adapted from https://ala.org.au)

With over 2000 extant species in 33 families, the order Rodentia is the most diversified mammalian order [11]. Within the Rodentia, the family Muridae is highly speciose, with 730 currently recognized species in 150 genera distributed in Eurasia, Africa and Australia [11]. Sixty-three native rodent species (including extinct species) have been recorded in Australia; all of them are in the subfamily Murinae [11–13]. Australian native rodents originated in southern Asia and colonized Australia in two main migration events, known as the old endemics and the new endemics, respectively [12]. The old endemic murines began to colonize Australia at the end of the Miocene period, 5–8 million years ago (MYA); the new endemics arrived in Australia around 1–2 MYA [11–13]. In Australia, the old endemics have diverged into 13 genera with 56 species [11–13] and colonized a wide range of terrestrial environments including arid areas, forests, wet open woodlands, swamps, waterways and grasslands from tropical to middle latitudes and from sea level to mountain peaks [13]. The new endemics have diverged into seven species, all in the genus *Rattus*: bush rat, *R. fuscipes* (Waterhouse, 1839), swamp rat, *R. lutreolus* (Gray, 1841), cane field rat, *R. sordidus* (Gould, 1858), Cape York rat, *R. leucopus* (Gray, 1867), long-haired rat, *R. villosissimus* (Waite, 1898), dusky rat, *R. colletti* (Thomas, 1904) and pale field rat, *R. tunneyi* (Thomas, 1904). Three of the seven new endemic species inhabit forests (e.g. rainforest, coastal forest and eucalypt forest); the others occur in open grasslands or arid environments [13–15] (Fig. 1). The seven native *Rattus* species in Australia are divided into two phylogenetic groups: the Australian group with six species and the New Guinean group with only *R. leucopus* [16].

Thirteen species of blood-sucking lice have been recorded from 12 species of endemic rodents in Australia [10, 17–21]. All of the 13 species are in the genus *Hoplopleura* (Hoplopleuridae) except for *P. spinulosa*, which was introduced into Australia with the brown rat, *R. norvegicus*, and the black rat, *R. rattus* [22]. Wang et al. [10] reported recently that *P. spinulosa* has switched to the endemic *R. villosissimus* in Australia. In this study, we investigated further the host expansion of *P. spinulosa* and two other introduced sucking lice, *Polyplax serrata* (Burmeister, 1839) and *Hoplopleura pacifica* Ewing, 1924, among endemic rodents in Australia. We show that *P. spinulosa* has expanded its host range to at least six endemic rodent species (including *R. villosissimus*) in Australia; *P. serrata* and *H. pacifica* however, have not expanded to any endemic rodents. The host expansion of *P. spinulosa* in Australia has occurred within a short time frame of 200 to 400 years, which is remarkably rapid relative to its host expansion in Eurasia where the species originated.

## Methods

### Collection of sucking lice and morphological examination

Sucking lice were collected from ethanol-preserved rodent specimens in seven museums across Australia (Table 1). Lice were dislodged from the host pelage with a head louse comb using a modified “cocktail shaking” technique [21]. First, rodents were put on a tray, one at a time; the pelage was gently and thoroughly combed with a head louse comb. Then, the rodent was transferred into a jar, which was filled with 80% ethanol, capped and gently shaken for ~ 1 min to dislodge lice from the host pelage. The rodent was then removed from the jar. Finally, the ethanol solution was filtered through a fine mesh and the filtrate was examined under a dissecting microscope (Nikon SMZ800N, Tokyo, Japan) for lice. Any lice present were removed from the mesh, kept in labelled individual vials (one per host specimen), filled with ethanol and stored at − 20 °C. For morphological examination, louse specimens were mounted on microscope slides in Canada balsam: intact specimens with minimal gut contents were selected and cleared in KOH (20%) and then sequentially immersed in acetic acid (10%), acid fuchsin (1%), ethanol (40%, 70% and 100%), pure clove oil and finally mounted on slides [23]. Louse specimens were examined and measured with a photomicroscope (Nikon ECLIPSE T*s* 2, Tokyo, Japan). The following distinct features were examined for the identification of *P. spinulosa*: the 3rd segment of antenna of the male and female; the shape of the thoracic sternal plate and the shape and setation of the paratergal plates. Descriptive format and abbreviations follow Kim et al. [24].

**Table 1:** Collections of sucking lice from murine rodent specimens in seven museums across Australia

Rodent species (*n* = 54)	No. of rodents sampled (*n* = 989)							Rodents with sucking lice	Rodents with *Polyplax* lice	No. of all sucking lice collected		No. of *Polyplax spinulosa* collected	
	QM	MV	AM	WAM	SA	NT	QVM			Adults	Nymphs	Adults	Nymphs
*Conilurus penicillatus*	–	–	–	4	7	11	–	6	–	24	50	–	–
*Hydromys chrysogaster*	–	–	6	–	–	–	5	3	–	24	11	–	–
*Leggadina forresti*	20	25	–	–	–	–	–	16	1	37	104	1	–
*Leggadina lakedownensis*	13	–	–	10	–	13	–	7	–	44	26	–	–
*Leporillus conditor*	–	1	–	–	3	1	–	3	–	29	16	–	–
*Mastacomys fuscus*	–	–	2	–	–	–	–	2	–	74	30	–	–
*Melomys burtoni*	1	–	–	–	–	5	–	4	–	9	7	–	–
*Melomys capensis*	–	–	–	–	3	–	–	–	–	20	13	–	–
*Melomys cervinipes*	25	–	–	–	–	–	–	22	–	121	–	–	–
*Mesembriomys gouldi*	3	6	–	–	–	–	–	3	–	30	34	–	–
*Mesembriomys macrurus*	–	1	–	12	4	3	–	3	1	11	7	1	–
*Notomys alexis*	–	29	7	–	–	–	–	8	–	11	7	–	–
*Notomys aquilo*	–	–	1	–	–	6	–	–	–	–	–	–	–
*Notomys cervinus*	–	6	4	–	10	–	–	5	–	32	18	–	–
*Notomys fuscus*	–	21	–	–	11	–	–	8	–	13	16	–	–
*Notomys longicaudatus*^a^	–	22	–	–	1	–	–	6	–	6	6	–	–
*Notomys mitchelli*	–	20	–	13	7	–	–	6	–	16	22	–	–
*Pseudomys albocinereus*	–	–	–	30	–	–	–	3	–	18	39	–	–
*Pseudomys apodemoides*	–	–	–	–	19	–	–	10	–	26	7	–	–
*Pseudomys australis*	–	27	–	–	–	–	–	14	–	36	11	–	–
*Pseudomys bolami*	–	–	11	–	–	–	–	3	–	13	1	–	–
*Pseudomys calabyi*	–	–	–	9	–	18	–	1	–	–	1	–	–
*Pseudomys chapmani*	–	–	–	20	2	–	–	2	–	1	2	–	–
*Pseudomys delicatulus*	28	–	14	–	–	–	–	9	–	16	12	–	–
*Pseudomys desertor*	5	–	–	–	–	–	–	4	–	20	35	–	–
*Pseudomys fieldi*	–	–	–	20	3	–	–	–	–	–	–	–	–
*Pseudomys gracilicaudatus*	13	–	5	–	–	–	–	7	–	11	28	–	–
*Pseudomys hermannsburgensis*	2	–	–	9	–	–	–	6	–	29	7	–	–
*Pseudomys higginsi*	–	–	–	–	–	–	6	4	–	4	7	–	–
*Pseudomys laborifex*	–	–	–	14	–	25	–	4	–	5	6	–	–
*Pseudomys nanus*	–	–	–	–	–	12	–	5	–	66	32	–	–
*Pseudomys novaehollandiae*	5	–	19	–	–	–	4	8	–	17	6	–	–
*Pseudomys occidentalis*	–	–	–	14	–	–	–	3	1	119	29	47	15
*Pseudomys patrius*	19	–	–	–	–	–	–	1	–	1	–	–	–
*Pseudomys shortridgei*	–	–	–	11	4	–	–	–	–	–	–	–	–
*Uromys caudimaculatus*	–	–	3	–	–	–	–	–	–	–	–	–	–
*Uromys hadrourus*	1	–	–	–	–	–	–	1	–	2	4	–	–
*Xeromys myoides*	14	–	–	–	–	1	–	3	–	2	3	–	–
*Zyzomys argurus*	–	–	–	–	–	9	–	3	–	9	5	–	–
*Zyzomys maini*	–	–	–	–	–	3	–	–	–	–	–	–	–
*Zyzomys palatalis*	–	–	–	–	–	9	–	3	–	12	15	–	–
*Zyzomys pedunculatus*	–	11	–	–	1	–	–	4	–	3	7	–	–
*Zyzomys woodwardi*	–	–	–	11	2	–	–	1	–	–	1	–	–
*Rattus colletti*	9	–	–	–	–	13	–	7	1	38	79	1	–
*Rattus fuscipes*	37	–	2	–	–	–	–	21	6	75	28	53	20
*Rattus leucopus*	27	–	6	5	–	–	–	16	–	23	51	–	–
*Rattus lutreolus*	8	–	5	–	–	–	7	12	4	52	23	12	13
*Rattus sordidus*	15	–	6	–	–	–	–	18	10	52	15	24	9
*Rattus tunneyi*	24	–	–	–	–	3	–	27	15	99	60	39	23
*Rattus villosissimus*	27	–	–	–	–	–	–	27	17	826	109	706	45
*Rattus rattus*^b^	2	7	–	–	–	–	–	5	5	35	73	35	73
*Mus musculus*^b^	1	–	–	–	–	6	–	1	–	–	1	–	–
*Rattus exulans*^b^	–	1	–	–	–	–	–	0	–	–	–	–	–
*Rattus norvegicus*^b^	–	–	–	–	–	3	–	0	–	–	–	–	–
Total	299	177	91	182	77	141	22	337	60	2111	1064	919	198

^a^Extinct species

^b^Introduced species to Australia

*Abbreviations*: QM, Queensland Museum; MV, Museums Victoria; AM, Australian Museum; WAM, Western Australian Museum; NTM, Museum and Art Gallery of the Northern Territory; QVM, Queen Victoria Museum and Art Gallery; SA, South Australian Museum

*Note*: “–” indicates no specimens were checked or collected

### DNA extraction, amplification and sequence analysis

Total DNA was extracted from individual louse specimens with DNeasy Tissue and Blood Kit (Qiagen, Hilden, Germany), following manufacturer protocols. We extracted DNA using a non-grinding method [25, 26] (Table 2). After DNA extraction, louse exoskeletons were mounted on microscope slides and examined morphologically. A fragment of the mitochondrial *cox*1 gene (~ 600 bp) was amplified by polymerase chain reaction (PCR) with primers mtd6 (5′-GGA GGA TTT GGA AAT TGA TTA GTT CC-3′) and mtd11 (5′-ACT GTA AAT ATA TGA TGA GCT CA-3′) [27]. A fragment of the mitochondrial *rrnL* gene (~ 320 bp) was amplified with primers 16SF (5′-TTA ATT CAA CAT CGA GGT CGC AA-3′) and Lx16SR (5′-GAC TGT GCT AAG GTA GCA TAA T-3′) [28]. PCR conditions were: initial step of 1 min at 94 °C, followed by 40 cycles of 10 s at 98 °C, 5 s at 40 °C (for *cox*1) and 52 °C (for *rrnL*) and 5 s at 72 °C and a final extension for 30 s at 72 °C. These primers target highly conserved sequence motifs among arthropods. PCR amplifications were 25 µl each using 12.5 µl of PrimeSTAR Max Premix Mix (Takara, Shiga, Japan), 9.5 µl H_2_O, 1 µl of each primer and 1 µl of DNA template. PCR products were purified using Wizard® SV Gel Clean-Up System (Promega, Madison, USA), following manufacturer instructions. Purified *cox*1 and *rrnL* amplicons were sequenced in both directions with the Sanger method at the Australian Genome Research Facilities (AGRF) in Brisbane, Australia. Sequence reads were assembled using Geneious 11.0.2; gene identities were verified by BLAST searches of GenBank.

**Table 2:** Murine rodent specimens (*n* = 63) from which *Polyplax spinulosa* was collected

Host species	Sample ID	Storage	Locality of collection	*Polyplax spinulosa* collected [*n* = 932 (201)^a^]	*Hoplopleura* spp. collected [*n* = 33 (26)^a^]	Collection date	Museum or collector
*Leggadina forresti*	JM4346	Spirit	Benditoota Waterhole, Queensland (25°37′S, 139°48′E)	1 (0)	0	28-Sep-1982	Queensland Museum
*Mesembriomys macrurus*	C7597	Spirit	Balanbrinni Ck, W Coast, Gulf of Carpentaria inland from Macarthur River, Northern Territory (16°58′S, 135°32′E)	1 (0)	0	No record	Museums Victoria
*Pseudomys occidentalis*	M43324	Spirit	Bluff Knoll, Western Australia (34°22′00″S, 118°15′00″E)	47 (15)	0	28-Sep-1994	Western Australian Museum
*Rattus colletti*	J21881	Spirit	Northern Territory	1 (0)	0	No record	Queensland Museum
*Rattus fuscipes*	J2722	Spirit	Brisbane, Fortitude Valley, Queensland (27°28′S, 153°2′E)	1 (3)	0	No record	Queensland Museum
	J2769	Spirit	Brisbane, Fortitude Valley, Queensland (27°28′S, 153°2′E)	1 (0)	0	No record	Queensland Museum
	J20068	Spirit	Gallangowan, Queensland (26°26′S, 152°20′E)	1 (1)	0	1-Aug-1955	Queensland Museum
	J20084	Spirit	Euramoo Ck, Danbulla, NE Atherton, Queensland (17°9′S, 145°37′E)	1 (1)	0	26-Aug-1956	Queensland Museum
	J20113	Spirit	Walsh Camp, 11.2 km SW Atherton, Queensland (17°20′S, 145°25′E)	46 (15)	0	8-Aug-1956	Queensland Museum
	M31441	Spirit	Comerong Is, Nowra, New South Wales (34°53′37″S, 150°44′56″E)	3 (0)	0	25-Jan-1995	Australian Museum
*Rattus lutreolus*	RLA^b,c,d^	Frozen	Chuwar Ipswich, Queensland (27°22′53.71″S, 152°47′12.72″E)	4 (3)	0	No record	Queensland Museum
	JM12492	Spirit	Camira, Queensland (27°37′S, 152°56′E)	2 (0)	0	12-May-1998	Queensland Museum
	JM12711	Spirit	Tinaroo Dam, Queensland (17°10′S, 145°33′E)	4 (10)	0	20-Feb-1963	Queensland Museum
	JM12771	Spirit	Boonah Shire, Mt Barney NP, Cronan Ck, Queensland (28°18′20″S, 152°41′25″E)	2 (0)	0	5-Oct-1993	Queensland Museum
*Rattus rattus*	Z65055^b,c,d^	Spirit	Victoria Range Road, 1.1 km WNW (296.7°) of intersection of Victoria Range Road and Sawmill Track, Grampians National Park, Victoria	9 (15)	0	Nov-2017	Museums Victoria
	RS92^b,c,e^	Spirit	Tuaran, Sabah (Borneo)	9 (0)	0	1-Mar-2008	Konstans Wells
	C28523	Spirit	Yarra Valley Metropolitan Park, Victoria	22 (57)	0	26-Jul-1990	Museums Victoria
	C36839	Spirit	12 Leura Ave, Rosanna, Victoria	1 (0)	0	20-Jun-2011	Museums Victoria
	C37126	Spirit	Cooinda Burrong Scout Camp, Grampians National Park, Victoria	2 (0)	0	22-Nov-2012	Museums Victoria
	Z65054	Spirit	Victoria Range Road, 1.1 km WNW (296.7°) of intersection of Victoria Range Road and Sawmill Track, Grampians National Park, Victoria	1 (1)	0	Nov-2017	Museums Victoria
	RS361^b,c,d^	Fresh	Australian Zoo Wildlife Hospital, Queensland	4 (3)	0	9-Aug-2019	Renfu Shao, Yalun Dong
*Rattus sordidus*	M34647	Spirit	Queensland	1 (1)	0	2-Mar-2000	Australian Museum
	J20061	Spirit	Walsh Camp, 11.2 km SW Atherton, Queensland (17°20′S, 145°25′E)	4 (2)	0	13-Aug-1956	Queensland Museum
	J20062	Spirit	Walsh Camp, 11.2 km SW Atherton, Queensland (17°20′S, 145°25′E)	1 (0)	0	8-Aug-1956	Queensland Museum
	JM4093	Spirit	York Downs, Queensland (12°45′11″S, 142°18′36″E)	1 (0)	0	14-May-1981	Queensland Museum
	JM13985	Spirit	Janie Ck Mth, 12 km SW of Cullen Point, N of Weipa, Queensland (12°2′13″S, 141°49′26″E)	1 (0)	0	9-Sep-1981	Queensland Museum
	JM13986	Spirit	Paperbark Flats, NW of Weipa, Queensland (12°6′33″S, 142°21′52″E)	1 (0)	0	7-Sep-1981	Queensland Museum
	JM15192	Spirit	Innisfail, Queensland (17°32′55″S, 145°51′02″E)	0 (1)	0	10-Sep-2002	Queensland Museum
	JM15199	Spirit	Innisfail, Queensland (17°32′55″S, 145°51′02″E)	12 (2)	0	10-Sep-2002	Queensland Museum
	JM23200	Spirit	Queensland	2 (3)	0	No record	Queensland Museum
	JM23201	Spirit	Queensland	1 (0)	0	No record	Queensland Museum
*Rattus tunneyi*	N19187^b,c^	Frozen	Mockers RD, Fernvale, Queensland (27°29′S, 152°40′E)	10 (0)	0	21-Feb-2010	Queensland Museum
	JM8136	Spirit	Leynora Downs, 25 km S of Rolleston, Queensland (24°38′S, 148°50′E)	1 (0)	0	24-Aug-1990	Queensland Museum
	JM12645	Spirit	Sunrise Hstd, NW Injune, Queensland (25°20′6″S, 148°5′47″E)	1 (0)	0	7-Oct-1996	Queensland Museum
	JM13324	Spirit	Maryborough, 2km from Maryborough & Hervey Bay Rd, Queensland (25°32′S, 152°42′E)	1 (0)	0	18-Aug-1999	Queensland Museum
	JM13785	Spirit	Northern Downs District, 15 km E Dalby, Queensland (27°9′24″S, 151°27′50″E)	0 (3)	0	18-Mar-1999	Queensland Museum
	JM13786	Spirit	Northern Downs District, 15 km E Dalby, Queensland (27°1′44″S, 151°15′59″E)	1 (0)	0	18-Mar-1999	Queensland Museum
	JM13788	Spirit	Northern Downs District, 15 km E Dalby, Queensland (27°1′44″S, 151°15′59″E)	2 (0)	0	18-Mar-1999	Queensland Museum
	J9201	Skin	Gallangowan, Queensland (26°26′S, 152°20′E)	2 (0)	0	No record	Queensland Museum
	JM1333	Spirit	Kilcoy area, Queensland (26°57′S, 152°34′E)	1 (8)	1 (0)	7-Nov-1973	Queensland Museum
	JM4102	Skin	Red Beach, 8 km S Cullen Point, Queensland (12°1′5″S, 141°53′55″E)	1 (0)	1 (0)	8-Sep-1980	Queensland Museum
	JM6923	Spirit	Cecil Plains, Brisbane, Queensland (27°32′S, 151°11′E)	3 (4)	0	1989	Queensland Museum
	JM7265	Spirit	Blue Lagoon, Moreton Is, W side, Queensland (27°6′S, 153°26′E)	1 (1)	0	6-Apr-1973	Queensland Museum
	J9204	Skin	Gallangowan, Queensland (26°26′S, 152°20′E)	12 (3)	0	No record	Queensland Museum
	J21294	Skin	Archookoora, via Kingaroy, Queensland (26°44′S, 151°48′E)	0 (1)	0	Oct-1969	Queensland Museum
	J22596	Skin	Brookfield, Gold Ck Rd, Brisbane, Queensland (27°30′S, 152°55′E)	3 (3)	0	26-May-1972	Queensland Museum
*Rattus villosissimus*	RVA	Frozen	Crossroads on Tonkoro Road, Queensland (24°08′56.6″S, 143°35′01.6″E)	30 (0)	0	9-Mar-2011	Queensland Museum
	RVB^b,c,d^	Frozen	Noonbah Station, Homestead, Queensland (24°06′27″S, 143°11′10″E)	260 (0)	0	31-Jul-2011	Queensland Museum
	RVC	Frozen	Queensland (24°17′41.5″S, 143°19′48.1″E)	150 (0)	0	2-Mar-2011	Queensland Museum
	RVD	Frozen	Noonbah Station, Homestead, Queensland (24°06′27″S, 143°11′10″E)	20 (0)	0	10-Apr-2011	Queensland Museum
	RVE	Frozen	Noonbah Station, Homestead, Queensland (24°06′27″S, 143°11′10″E)	3 (9)	0	No record	Queensland Museum
	RVF	Frozen	West Queensland, Queensland	1 (0)	0	No record	Queensland Museum
	RVH	Frozen	Vergemont, Queensland (24°06′26.0″S, 143°11′10.8″E)	23 (12)	0	15-Apr-2011	Queensland Museum
	RVI	Frozen	West Queensland, Queensland	120 (0)	0	No record	Queensland Museum
	RVJ	Frozen	Noonbah Station, Homestead, Queensland (24°06′27″S, 143°11′10″E)	0 (5)	0	No record	Queensland Museum
	RVK	Frozen	West Queensland, Queensland	12 (2)	0	28-Apr-2011	Queensland Museum
	RVL	Frozen	1 km east of Waterloo Bore PD Dam, Queensland (24°09′58.9″S, 143°14′58.8″E)	50 (0)	0	24-Feb-2011	Queensland Museum
	RVM	Frozen	Thomson River 200 m west of bridge, Queensland (24°05′45.5″S, 143°22′55.2″E)	8 (0)	0	19-Apr-2011	Queensland Museum
	N68645	Frozen	Glenore Vena Park Rd. Normanton, Queensland (18°17′32.15″S, 141°12′41.85″E)	1 (0)	0	11-Jun-2011	Queensland Museum
	JM4824	Spirit	Sandringham (61–22), Montara Dune, Queensland (23°56′S, 138°47′E)	7 (3)	0	28-Jul-1984	Queensland Museum
	JM4825	Spirit	Sandringham (61–22), Montara Dune, Queensland (23°56′S, 138°47′E)	12 (8)	17 (13)	29-Jul-1984	Queensland Museum
	JM5234	Spirit	Marked Tree Waterhole, 2 km North, Queensland (23°17′S, 138°9′E)	1 (0)	10 (7)	8-Aug-1985	Queensland Museum
	JM10742	Spirit	Diamantina Lakes, Queensland (23°40′S, 141°5′E)	8 (6)	4 (6)	10-14 Aug-1981	Queensland Museum

^a^No. of adult specimens outside parenthesis, number of nymphal specimens inside parenthesis

^b^*Polyplax spinulosa* specimens from which DNA extraction was successful

^c^Mitochondrial *rrnL* gene sequenced

^d^Mitochondrial *cox*1 gene sequenced

^e^The only rodent specimen collected outside Australia in the present study

### Phylogenetic analysis of *rrnL* gene fragments

Multiple sequence alignments were created with Geneious 11.0.2 software [29]. An unrooted neighbor-joining (NJ) consensus tree of *rrnL* gene fragments was constructed using the distance matrix calculated by the Kimura-Nei model of evolution as implemented in the Geneious 11.0.2 [29]. A bootstrap analysis using 1000 replicates was performed on the resulting tree for node support.

## Results

### New hosts of *Polyplax spinulosa* among endemic rodents in Australia

We examined 989 rodent specimens of 54 species (50 endemic species, four introduced species) in seven museums across Australia, and collected 2111 adult sucking lice and 1064 nymphal sucking lice (Table 1). We also examined 13 adult sucking lice and three nymphal sucking lice collected from two *R. rattus* specimens from Sabah (Borneo) and Sunshine Coast (Queensland), respectively (Table 2). We did not examine specimens of the other 13 endemic rodent species (nine of them extinct) due to the unavailability of specimens to us. Of the 2124 adult sucking lice collected, 932 specimens (i.e. 43.9%) were identified as *Polyplax spinulosa*; the remaining specimens were *Hoplopleura* spp., some of which represent undescribed species. *Polyplax spinulosa* was found on 63 individual rodents, i.e. 6.4% of the total number of rodents we examined (*n* = 991); these 63 individual rodents were collected over a period of 65 years (1955–2019) from three old endemic species, six new endemic species and one introduced species (Table 2). The number of *P. spinulosa* we collected varied substantially among the 10 host species. A single adult *P. spinulosa* was collected from the old endemic species, *Leggadina forresti* and *Mesembriomys macrurus*, respectively; another single adult *P. spinulosa* was collected from the new endemic species, *R. colletti* (Table 2). Twelve to 706 adults of *P. spinulosa* were collected from each of the other six old or new endemic species, together with 9 to 73 *Polyplax* nymphs. Forty-eight adult specimens of *P. spinulosa* were collected from the introduced black rat, *R. rattus*, together with 76 *Polyplax* nymphs (Table 2). The intensity of *P. spinulosa* presence also varied substantially from one adult louse to 260 adult lice on an individual host rodent (Table 2). Of the 63 individual rodents that were parasitized by *P. spinulosa*, five rodents also hosted *Hoplopleura* spp., giving a double-infestation rate of 8.2%; in each case of double-infestation, the abundance was similar between *P. spinulosa* and *Hoplopleura* spp. (Table 2).

We collected a single sucking louse from the introduced house mouse, *Mus musculus* (*n* = 7, Table 1), but could not identify it to either genus or species level as the specimen was an early-stage nymph. We did not find *P. spinulosa* on the other 43 endemic rodent species (*n* = 716), or on the other two introduced species: the brown rat, *R. norvegicus* (*n* = 3) and the Polynesian rat, *R. exulans* (*n* = 1). We did not find the other two species of introduced sucking lice, *P. serrata* and *H. pacifica*, on any of the 991 rodent specimens we checked (Tables 1, 2).

### Morphology of *Polyplax spinulosa* recorded on endemic rodents in Australia

To confirm the identification of *P. spinulosa*, we mounted 30 adult sucking louse specimens (16♂, 14♀) on microscope slides and examined their morphology in detail; these 30 specimens were from 10 rodent hosts: ex *M. macrurus* (MV C7597, 1♀) (note: museum name abbreviation, rodent specimen registration number and number of louse specimen mounted and sex are listed hereafter), ex *L. forresti* (QM JM4346, 1♂), ex *P. occidentalis* (WAM M43324, 2♂, 1♀), ex *R. colletti* (QM J21881, 1♀), ex *R. fuscipes* (QM J20113, 2♂, 1♀), ex *R. lutreolus* (MV RLA, 2♂, 2♀), ex *R. sordidus* (QM J92310, 1♂, 1♀), ex *R. tunneyi* (QM N19187, 2♂, 2♀), ex *R. villosissimus* (QM RVB, 3♂, 2♀), ex *R. rattus* (MV Z65055, 2♂, 2♀), ex *R. rattus* (Sabah RS92, 1♂, 1♀) (note: MV for Museums Victoria, WAM for Western Australian Museum, QM for Queensland Museum) (Additional file 1: Figure S1, Additional file 2: Figure S2). We also examined all other unmounted specimens under a high-magnification binocular microscope. *Polyplax spinulosa* is morphologically distinct from other *Polyplax* species but only a few minor characters distinguish it from *P. serrata*, *P. wallacei* Durden, 1987 and *P. reclinata* (Nitzsch, 1864). We observed and relied on four distinct characters to identify *P. spinulosa* collected from rodent hosts. First, *P. spinulosa* has a shield shaped, six-sided thoracic sternal plate (Additional file 3: Figure S3), whereas *P. reclinata* has a flat anterior margin on this plate. *Polyplax serrata* is smaller than *P. spinulosa* in body length and its thoracic sternal plate has a rounded anterior margin [30]. The thoracic sternal plate of *P. wallacei* is uniquely shield shaped with extended anterolateral angles [31]. Secondly, the spiracles of the paratergal plates of *P. reclinata* are larger than those of *P. spinulosa*. Thirdly, the setae on the paratergal plates of *P. reclinata* are longer than in *P. spinulosa*. The ventral posterior seta on the third paratergal plate of *P. serrata* is much longer than the corresponding dorsal seta, whereas in *P. spinulosa* both of these setae are short and about equal in length [30]. Paratergal plates VI and VII of *P. wallacei* each have two long apical setae; the dorsal posterior seta on paratergal plates I to III is longer than the corresponding ventral seta [31]. Fourth, the posterior setae on paratergal plate IV are the same length or longer than paratergal plate IV in *P. serrata*, while in *P. spinulosa*, the posterior setae of each paratergal plate are shorter than each corresponding paratergal plate [32, 33]. Additionally, *P. reclinata* parasitizes shrews in Africa and Eurasia [7] and would not be expected to parasitize murine rodents or to occur in Australia.

### Two genotypes of *Polyplax spinulosa* revealed by mitochondrial *rrnL* gene sequences

**Fig. 2:**
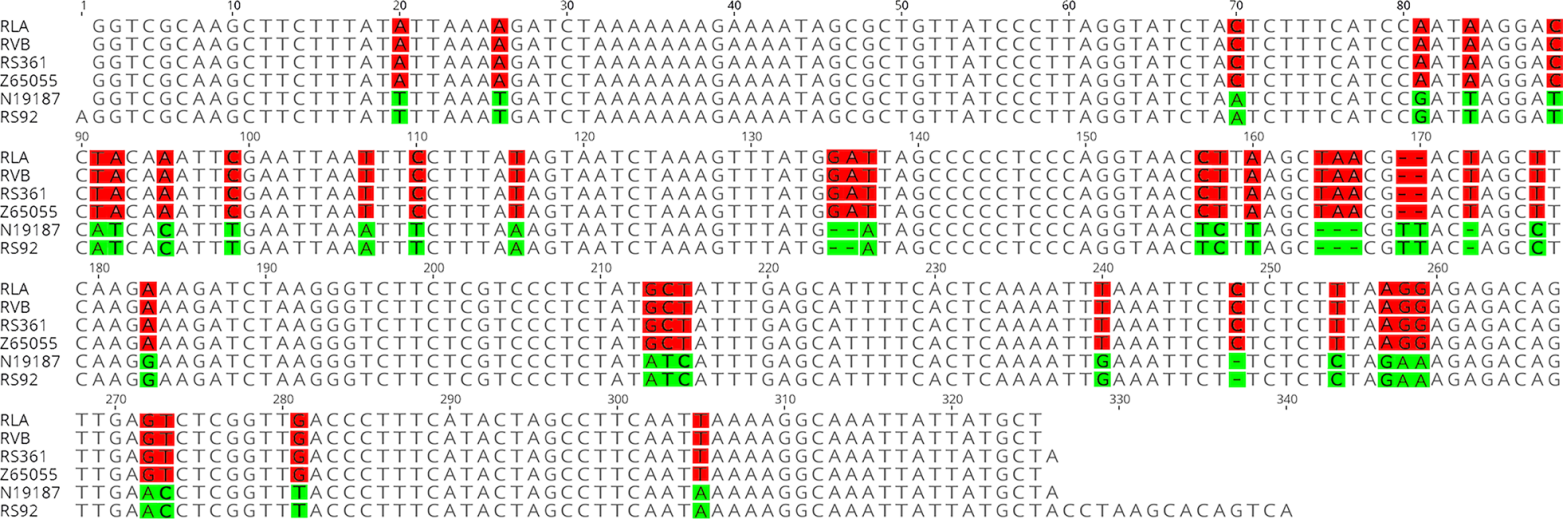
Sequence alignment of the mitochondrial *rrnL* gene of RLA, RVB, RS361, Z65055, RS92 and N19187. Red and green shading indicates nucleotide variation between the two genotypes

**Fig. 3:**
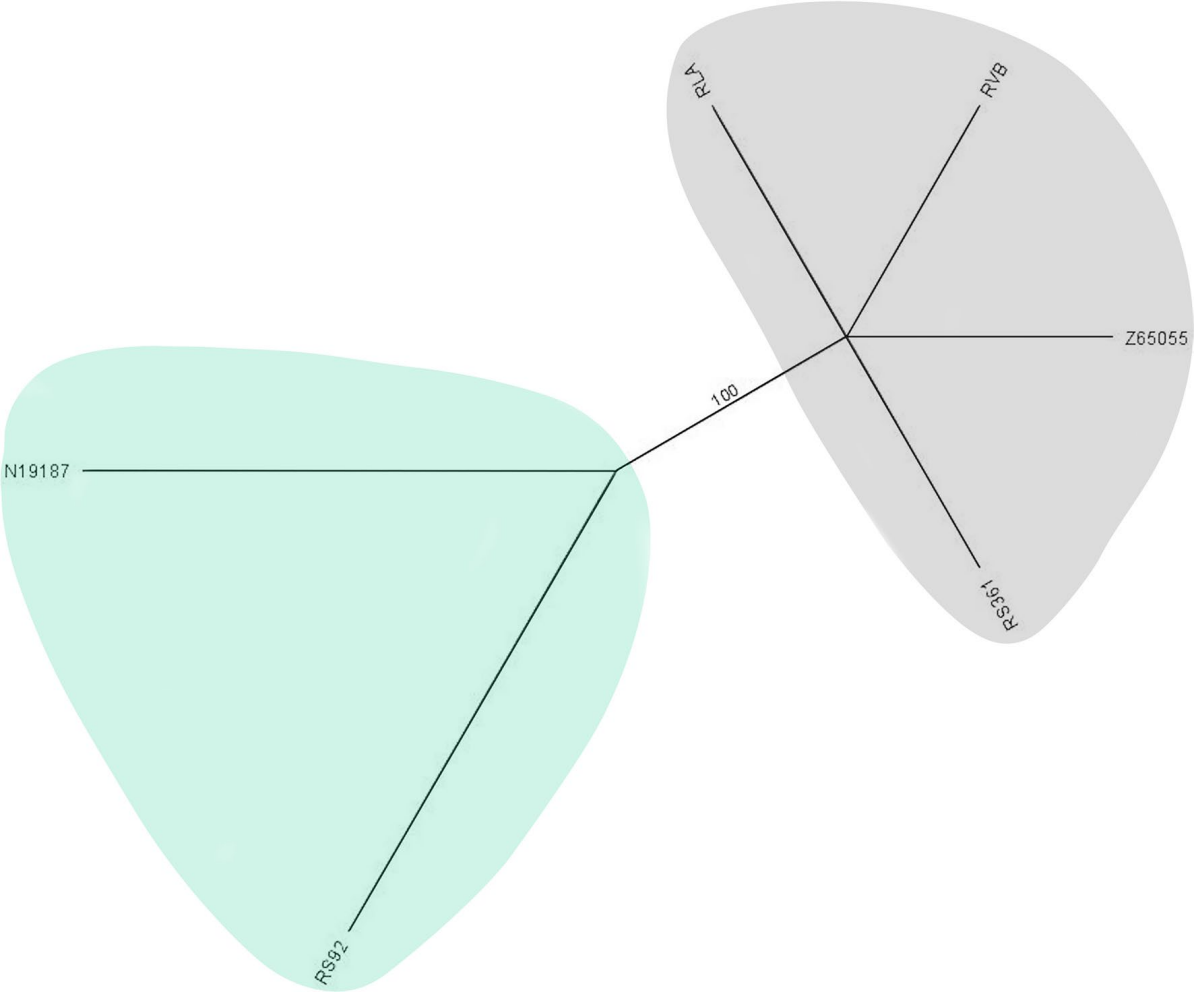
An unrooted neighbour-joining tree based on *rrnL* sequences of six *Polyplax spinulosa* specimens showing two genotypes (see also Fig. 5). Bootstrap value for the main branch is shown

The vast majority of the *P. spinulosa* specimens we collected in museums were old; their hosts had been fixed in formalin prior to preservation in ethanol. Therefore, most lice collected from these hosts were not suitable for molecular analysis. Nevertheless, we sequenced successfully the mitochondrial *rrnL* gene fragment (~320 bp) of six *P. spinulosa* specimens and the mitochondrial *cox*1 gene fragment (~ 600 bp) of four *P. spinulosa* specimens (Table 2). Comparison of the *rrnL* sequences revealed two genotypes that differed by 7.5% (Figs. 2, 3). Genotype 1 was shared (100% identical) by four *P. spinulosa* specimens found on different *Rattus* species in Queensland and Victoria: *R. lutreolus* (QM RLA), *R. villosissimus* (QM RVB) and *R. rattus* (MV Z65055 and RS361 from Queensland), respectively. We also obtained the *cox*1 sequences of these four specimens, which had > 99.3% identity to each other and to the published *cox*1 sequence of *P. spinulosa* collected from *R. norvegicus* in the Czech Republic (GenBank: EU162140 [34]) (Additional file 4: Figure S4), indicating a European origin of Genotype 1. The Genotype 2 *rrnL* sequence was shared (100% identical) by two *P. spinulosa* specimens found on *R. tunneyi* from Queensland (QM N19187) and *R. rattus* (RS92) from Sabah (Borneo) (Table 2, Fig. 2), thus indicating a Southeast Asian origin of this genotype. We were unable to obtain a *cox*1 sequence from these two *P. spinulosa* specimens (QM N19187 and RS92) despite repeated attempts. Although we did not have data on the hosts, the two genotypes of *P. spinulosa* are likely related to the two forms of *R. rattus* in Australia: the Oceanic form that came with the First Fleet and the Asian form [35].

## Discussion

### Host expansion of *Polyplax spinulosa* has been extremely rapid in Australia compared to Eurasia

The old endemic rodents arrived on the Australian continent 5–8 MYA at the end of the Miocene period; the new endemics arrived around 1–2 MYA [11–13]. *Hoplopleura* appears to be the only genus of sucking lice carried into Australia by the old endemic rodents because all of the known sucking louse species that parasitize endemic rodents are in this genus except *P. spinulosa* [10]. The cosmopolitan spiny rat louse, *P. spinulosa*, was introduced into Australia with its two hosts, the brown rat, *R. norvegicus*, and the black rat, *R. rattus*, by European explorers and colonizers [22, 36]. The earliest record of a European ship landing in Australia was in 1606 [37, 38], followed by British settlement in 1788 [39]. Thus, *P. spinulosa* would have had 200 to 400 years to expand and adapt to new hosts in Australia. The presence of *P. spinulosa* on endemic *Rattus* species in settled areas in Australia was briefly mentioned by Calaby and Murray [36]; however, there were no follow-up studies to ascertain whether these *P. spinulosa* lice were stragglers or established ectoparasites on the new host species. Only introduced *R. norvegicus* and *R. rattus* have been recognized as hosts of *P. spinulosa* in Australia [22].

**Fig. 4:**
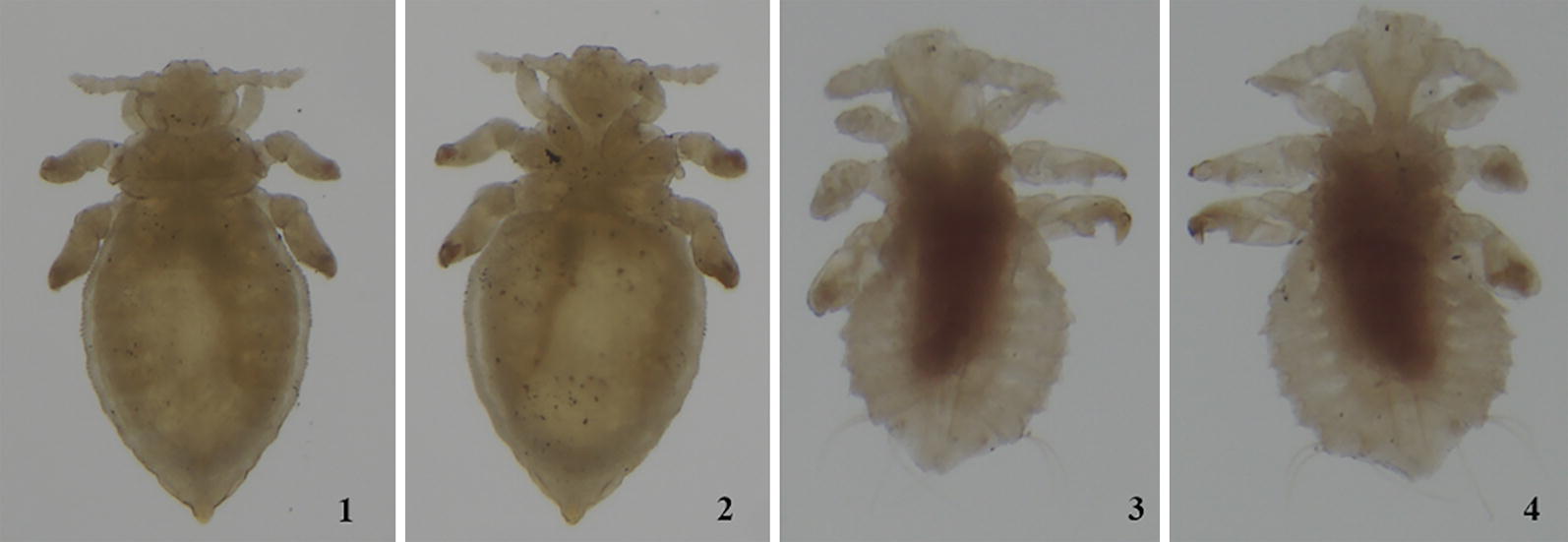
Uncleared nymphs of *Hoplopleura* sp. from *Notomys mitchelli*: (1) dorsal surface and (2) ventral surface. Uncleared nymphs of *Polyplax spinulosa*: (3) dorsal surface and (4) ventral surface

**Fig. 5:**
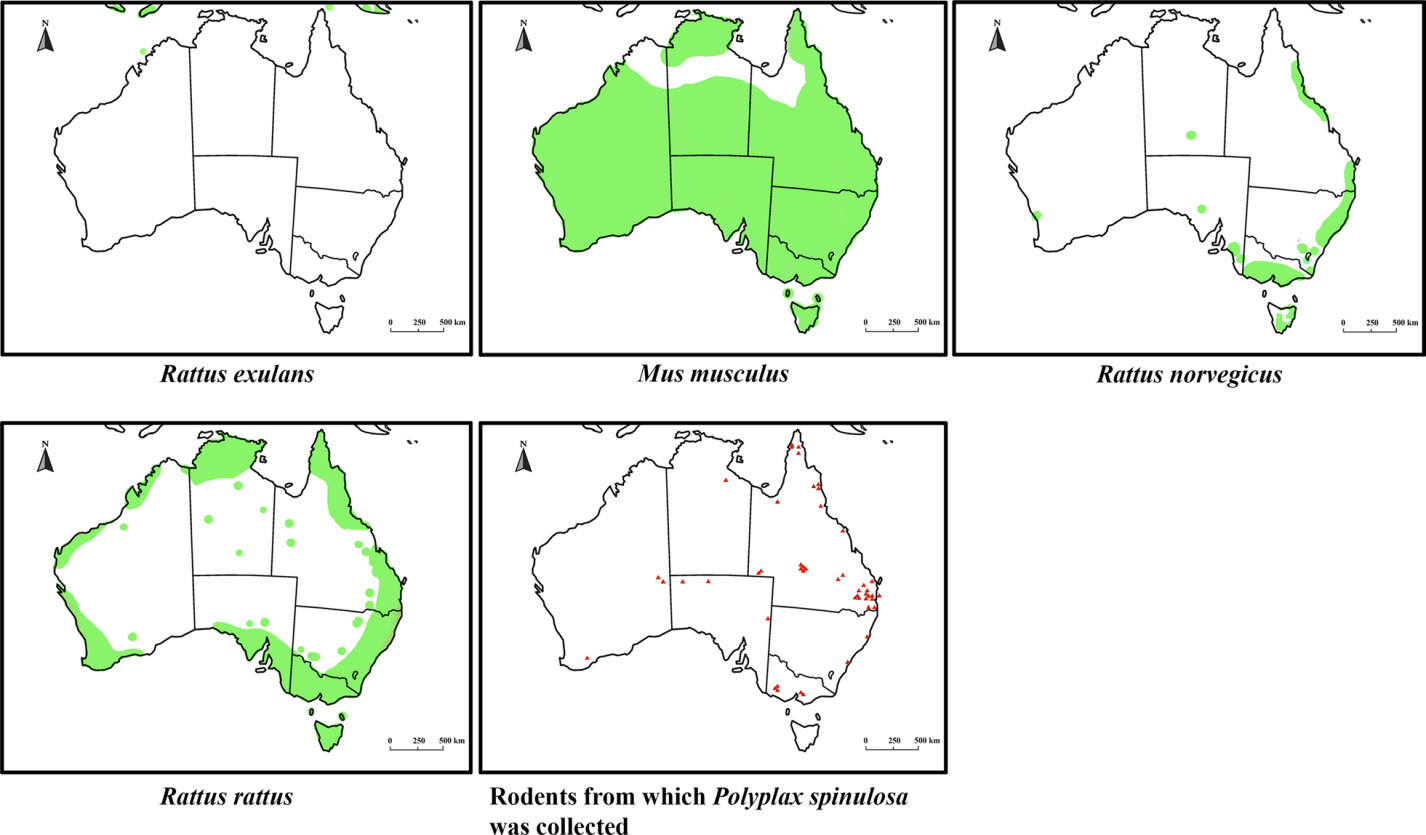
Distribution of introduced rodent species in Australia and locations of the rodents from which *Polyplax spinulosa* was collected in this study (adapted from https://ala.org.au)

Host switching by introduced sucking lice to endemic Australian rodents was reported only very recently. Wang et al. [10] showed for the first time that *P. spinulosa* had switched to, and established on, an endemic rodent, the long-haired rat, *R. villosissimus*. In the present study, we further investigated the host expansion by introduced sucking lice among the endemic rodents in Australia in more detail by examining 989 rodent specimens of 50 endemic species and four introduced species of murines deposited in seven museums across Australia. We also examined lice collected from two *R. rattus* specimens collected in Sabah (Borneo) and Sunshine Coast (Queensland), respectively. We found *P. spinulosa* on 63 individual rodents of three old endemic murine species and six new endemic murine species, in addition to the introduced host, *R. rattus* (Tables 1, 2). We cannot exclude the presence of *P. spinulosa* on the golden-backed tree rat, *Mesembriomys macrurus*, Forrest’s mouse, *Leggadina forresti* or the dusky rat, *Rattus colletti*, as stragglers as only a single *P. spinulosa* louse was found on each of these murine species (Tables 1, 2). However, *P. spinulosa* was much more abundant on the other six old and new endemic rodent species: 12 to 706 adults of *P. spinulosa* specimens were found on each of these rodent species. In addition to the adult *P. spinulosa*, 9 to 73 *Polyplax* nymphs were also found on each of these rodent species. These nymphs cannot be identified to the species level but can be identified to the genus *Polyplax* (Fig. 4). Furthermore, *P. spinulosa* was found on multiple rodent individuals (4 to 17) from different locations collected over decades for all of the six rodent species except for *Pesudomys occidentalis* (Tables 1, 2). The 63 rodent specimens from which we collected *P. spinulosa* are mostly in Queensland but are distributed in all other Australian states except for Tasmania (Fig. 5). Our data indicate strongly that *P. spinulosa* has expanded its host range to at least six endemic rodent species in Australia in the time frame of 200 to 400 years. Outside Australia, *P. spinulosa* is known to parasitize eight endemic rat species in Eurasia where it originated; one of its sister species, *P. serrata*, parasitizes 10 mouse species (9 *Apodemus* spp. and *Mus musculus*) [7]. The extant host ranges of *P. spinulosa* and *P. serrata* indicate that these two species likely diverged ~ 16 MYA when rats and mice diverged [40–42]. The two genotypes indicated by our limited *rrnL* sequence data also support an ancient origin of *P. spinulosa* in Eurasia. Therefore, the host range of *P. spinulosa* in Eurasia (i.e. 8 rat species) has taken millions of years to form. During this time period, *P. spinulosa* could have ample opportunities to switch and adapt to a much broader range of rodents (914 species in Eurasia) than in Australia (63 species) [43]. Apparently, the host expansion of *P. spinulosa* to at least six endemic murine species in Australia has occurred at a remarkably much faster pace than its host expansion in Eurasia. Furthermore, while *P. spinulosa* parasitizes only rats outside Australia, it expands to both endemic rats and an endemic mouse, *Pseudomys occidentalis*, in Australia.

### How did *Polyplax spinulosa* spread to its new hosts in Australia?

Host switching by sucking lice usually requires physical contact between hosts, e.g. living in communal nests/roosts, hosts breeding in close proximity, conspecific aggressive contact and during predator-prey contact [31]. Wang et al. [10] proposed that the wide distribution of the black rat, *R. rattus*, in Australia most likely facilitated the switch of *P. spinulosa* to the long-haired rat, *R. villosissimus*. This is also likely the case for the spread of *P. spinulosa* to the other endemic rodents revealed by the present study. The brown rat, *R. norvegicus*, and the Polynesian rat, *R. exulans*, are also hosts to *P. spinulosa* and are present in Australia as invasive species [22]. However, *R. exulans* is only found on a few offshore islands of Australia such as Adele Island and Norfolk Island but is absent from mainland Australia [12]. *Rattus norvegicus* inhabits primarily coastal urban areas of Australia, close to human populations [11]. *Rattus rattus*, however, is much more widely distributed than *R. norvegicus* and *R. exulans. Rattus rattus* can be found in coastal areas, in inland arid areas, on islands, in both human settlements or in areas with no human settlement in Australia (Fig. 5) [12]. Globally, *R. rattus* is one of the most successfully adapted invasive animal species and can be found on every continent except Antarctica [11]. The wide distribution of *R. rattus* would certainly create ample opportunities for it to have physical contacts with endemic rodents and for its parasites including *P. spinulosa* to transfer and adapt to new hosts. Overlapping distributions and physical contacts between endemic rodents could help *P. spinulosa* to expand its host range further once it had transferred from *R. rattus* to an endemic rodent species. All of the endemic rodent species on which we found *P. spinulosa* are abundant except for *Pseudomys occidentalis* and *Mesembriomys macrurus*, which are near threatened species on the ICUN Red List (https://www.iucnredlist.org/). Five of these species (*Leggadina forresti*, *R. fuscipes*, *R. lutreolus*, *Rattus tunneyi* and *R. villosissimus*) have wide distributions in Australia, which would facilitate the host expansion of *P. spinulosa*. In particular, during population explosions, *R. villosissimus* can be found over an area of 130,000 km^2^ in high density (thus the common name, plague rat) [13]. The wide distribution and population explosions of *R. villosissimus* would certainly generate plenty of opportunities to either pick up or pass on *P. spinulosa* to other rodents. On the other hand, the rodent species on which we did not find any *P. spinulosa* are either those with localised distributions such as *Rattus leucopus* and *Melomys capensis* (only in the Cape York area), those near threated, vulnerable or endangered such as *Pseudomys fieldi*, *Conilurus penicillatus*, *Leporillus conditor*, *Pseudomys fumeus* (https://www.iucnredlist.org/), or those with specialised habitats such as the water rat, *Hydromys chrysogaster*.

### Why did *Polyplax spinulosa* succeed in host expansions in Australia whereas *Polyplax serrata* and *Hoplopleura pacifica* failed?

In addition to *P. spinulosa*, two other species of sucking lice, *H. pacifica* and *P. serrata*, have also been introduced into Australia with their commensal rodent hosts [22]. Like *P. spinulosa*, *H. pacifica* was introduced into Australia with the black rat, *R. rattus*, whereas *P. serrata* was introduced into Australia with the house mouse, *M. musculus* [22]. Outside Australia, *H. pacifica* has been recorded from six *Rattus* species including *R. rattus*, and *P. serrata* has been recorded from nine Eurasian *Apodemus* mouse species and *M. musculus* [7]. In stark contrast to *P. spinulosa*, neither *H. pacifica* nor *P. serrata* was found on any of the rodent specimens we examined in the present study (Tables 1, 2).

Why did *P. spinulosa* expand its host range successfully whereas *P. serrata* and *H. pacifica* failed? Sucking lice (Anoplura) are the most host-specific ectoparasites, are wingless, and feed only on host blood; this specialised life style, in general, limits their ability to transfer to, and establish on, new hosts [2, 7, 44]. However, the host specificity of sucking lice varies from species to species. Of the 532 species of sucking lice listed by Durden and Musser [3], 316 louse species are found on only a single host species, 92 louse species on two host species, 42 louse species on three host species, and 82 louse species including *P. spinulosa*, *P. serrata* and *H. pacifica* on four or more host species [7]. In the cases where one species of sucking louse parasitizes multiple host species, these hosts are almost always closely related, often in the same genus. Host species availability, however, is not the only factor that determines host specificity of sucking lice. The present study indicates that factors specific to each louse species also play a major role in determining the host specificity of sucking lice. These specific factors may pertain to the genetics or ecology of the louse species or its hosts, or both. The failure of *H. pacifica* to expand its host range in Australia is clearly due entirely to its own genetics or ecology because *H. pacifica* shares the same host, *R. rattus*, with *P. spinulosa;* host factors, thus, can be excluded in this case. The failure of *P. serrata* is more likely due to host factors. *Polyplax serrata* and *P. spinulosa* are closely related congeneric species with very similar morphology [45] and display similar host specificity outside Australia: *P. serrata* parasitizes 10 species of mice (9 *Apodemus* spp. and *Mus musculus*) whereas *P. spinulosa* parasitizes eight species of rats (seven *Rattus* spp. and *Bandicota bengalensis*) [7]*. Polyplax serrata* and *P. spinulosa* were introduced into Australia by *M. musculus* and *R. rattus*, respectively, through European exploration and colonization [46]. These two *Polyplax* species would have an approximately equal time frame (i.e. 200–400 years) and an equal number of potential new hosts (i.e. 63 endemic mouse and rat species) on which to potentially expand. It is very likely that the ecology of *M. musculus* played a major role in the failure of *P. serrata* to expand its host range in Australia. In comparison to *R. rattus*, *M. musculus* is much more close to human settlements and less adaptable to utilizing environments without human settlements despite the fact that *M. musculus* is more widely distributed than *R. rattus* in Australia (Fig. 5) [13]. Thus, *M. musculus* would have significantly fewer opportunities for physical contacts with endemic rodents, which might have hampered the transfer of *P. serrata* to endemic rodents in Australia.

## Conclusions

We have shown that *P. spinulosa* has expanded its host range to at least six endemic rat and mouse species in Australia in the time frame of 200 to 400 years since it was introduced, which is extremely rapid relative to its host expansion to eight native rat species in Eurasia in ~ 16 millions of years since it diverged from *P. serrata*. The host expansion of *P. spinulosa* is remarkable for a blood-sucking louse, and is in stark contrast to the absence of host expansion of *P. serrata* and *H. pacifica* in Australia. Comparison among these three introduced sucking lice indicates that both louse-specific factors and host-specific factors have contributed to host expansion. The successful host expansion of *P. spinulosa* can be attributed to both its genetics and ecology, plus the ecology of *R. rattus*, which carried *P. spinulosa* into Australia. The failure of *H. pacifica* to expand its host range is entirely due to its genetics or ecology because it shares the same host, *R. rattus*, with *P. spinulosa*. The failure of *P. serrata*, however, is very likely due to the ecology of its host, *M. musculus*, which is limited usually to human settlement areas and may not have sufficient opportunities to transfer *P. serrata* to endemic rodents. We expect further detailed comparative studies among these introduced sucking lice may pinpoint the exact factors of genetics or ecology that determine host specificity and host adaptation of sucking lice.

## Supplementary information

**Additional file 1: Figure S1.** Male *Polyplax spinulosa* collected from: (1) *Rattus fuscipes*; (2) *Rattus lutreolus*; (3) *Rattus sordidus*; (4) *Rattus tunneyi*; (5) *Rattus villosissimus*; (6) *Rattus rattus*; (7) *Pseudomys occidentalis*; and (8) *Leggadina forresti* (note: the shape of the abdomen can vary depending on the amount of distention from previous blood meals and the action of clearing chemicals prior to slide-mounting).

**Additional file 2: Figure S2.** Cleared female *Polyplax spinulosa* from different species of murine rodents: (1) *Rattus colletti*; (2) *R. fuscipes*; (3) *R. lutreolus*; (4) *R. sordidus*; (5) *R. tunneyi*; (6) *R. villosissimus*; (7) *R. rattus*; (8) *Mesembriomys macrurus*; and (9) *Pseudomys occidentalis* (note, the outline of an egg can be seen in 1) (note: the shape of the abdomen can vary depending on the amount of distention from previous blood meals and the action of clearing chemicals prior to slide-mounting).

**Additional file 3: Figure S3.** Thoracic sternal plates of *Polyplax spinulosa* collected from different species of *Rattus*: (1) ♂ from *R. fuscipes*; (2) ♀ from *R. fuscipes*; (3) ♂ from *R. lutreolus*; (4) ♀ from *R. lutreolus*; (5) ♂ from *R. sordidus*; (6) ♀ from *R. sordidus*; (7) ♂ from *R. tunneyi*; (8) ♀ from *R. tunneyi*; (9) ♂ from *R. villosissimus*; (10) ♀ from *R. villosissimus*; (11) ♂ from *R. rattus*; and (12) ♀ from *R. rattus*.

**Additional file 4: Figure S4.** Sequence analysis of the mitochondrial *cox*1 gene of RLA, RVB, RS361 and Z65055 in comparison with EU162140. The four sites with nucleotide variation are indicated by red and green shading.

## Data Availability

Data supporting the conclusions of this article are included within the article. The gene sequences generated are available in the GenBank repository under the accession numbers MN193570-MN193577, MN427448 and MN434185. The mounted microscopic slides of *Polyplax spinulosa* have been deposited in museums in Australia. The registration numbers of slides are: PHT-4 and PHT-5 (Museums Victoria), E102080 and E102081 (Western Australian Museum) and T246563-T246573 (Queensland Museum).

## Abbreviations

MYA: million years ago
PCR: polymerase chain reaction
BLAST: Basic Local Alignment Search Tool
NJ consensus tree: neighbor-joining consensus tree
*cox*1: cytochrome *c* oxidase subunit 1
*rrnL*: 16S ribosomal RNA
AGRF: Australian Genome Research Facilities
MV: Museums Victoria
QM: Queensland Museum
WAM: Western Australian Museum
