# Supplementary figures and images for: Rapid host expansion of an introduced parasite, the spiny rat louse Polyplax spinulosa (Psocodea: Phthiraptera: Polyplacidae), among endemic rodents in Australia
Source: Parasit Vectors. 2020 Feb 18;13:83. doi: 10.1186/s13071-020-3957-y (PMC7029564; doi:10.1186/s13071-020-3957-y)

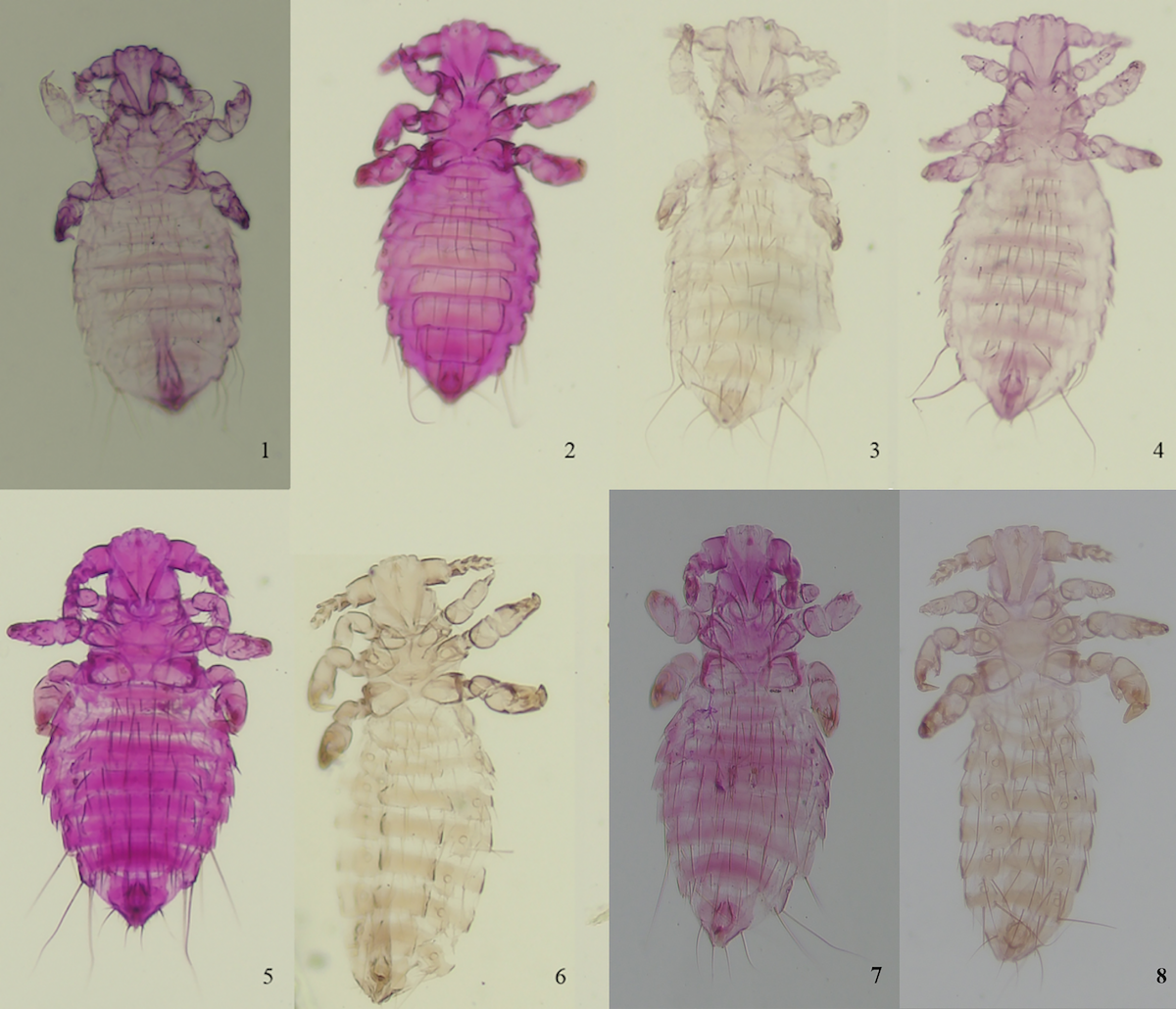

Supplement: Supplementary file 1 — Additional file 1: Figure S1. Male Polyplax spinulosa collected from: (1) Rattus fuscipes; (2) Rattus lutreolus; (3) Rattus sordidus; (4) Rattus tunneyi; (5) Rattus villosissimus; (6) Rattus rattus; (7) Pseudomys occidentalis; and (8) Leggadina forresti (note: the shape of the abdomen can vary depending on the amount of distention from previous blood meals and the action of clearing chemicals prior to slide-mounting). [file 13071_2020_3957_MOESM1_ESM.tif]

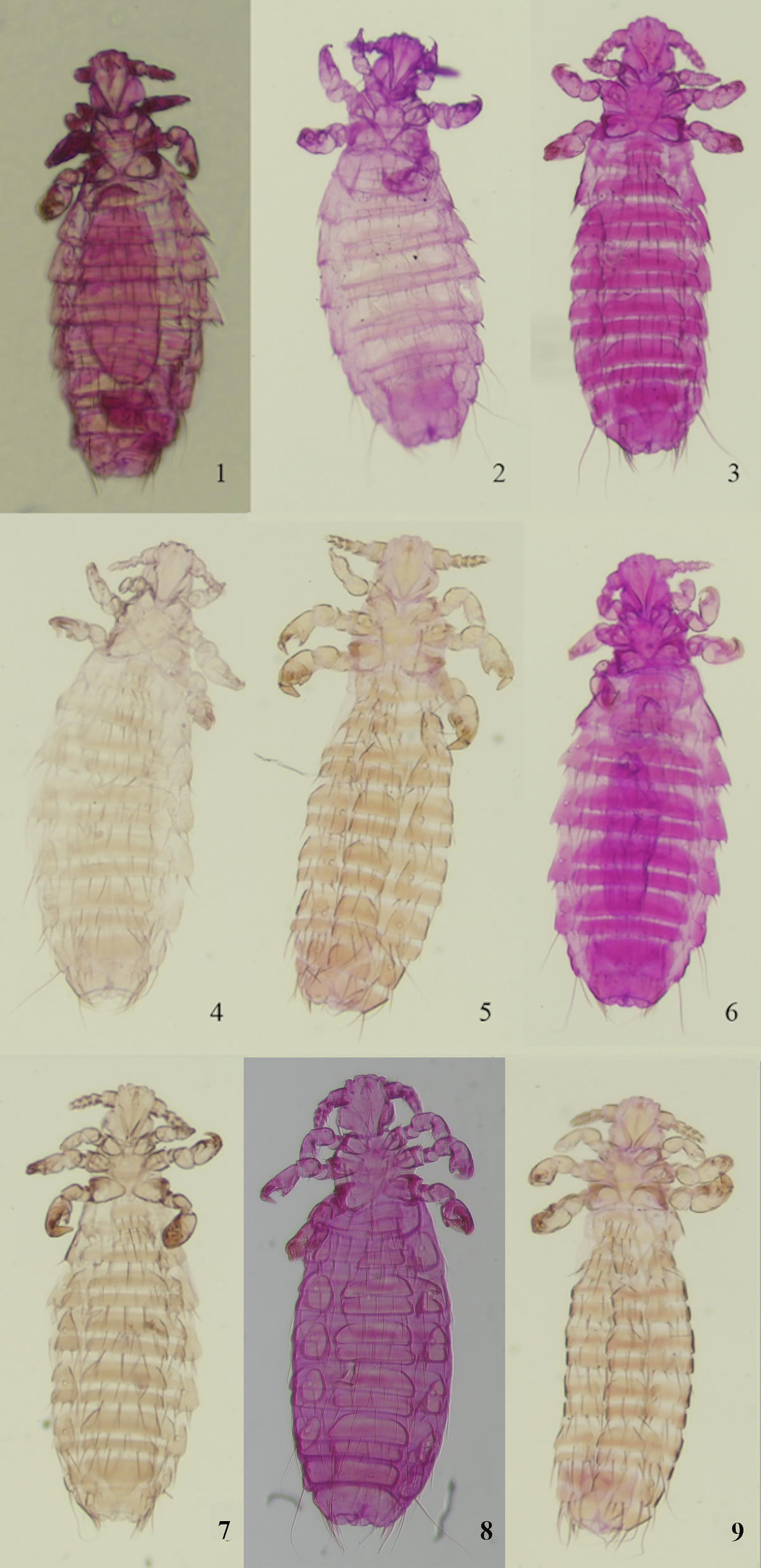

Supplement: Supplementary file 2 — Additional file 2: Figure S2. Cleared female Polyplax spinulosa from different species of murine rodents: (1) Rattus colletti; (2) R. fuscipes; (3) R. lutreolus; (4) R. sordidus; (5) R. tunneyi; (6) R. villosissimus; (7) R. rattus; (8) Mesembriomys macrurus; and (9) Pseudomys occidentalis (note, the outline of an egg can be seen in 1) (note: the shape of the abdomen can vary depending on the amount of distention from previous blood meals and the action of clearing chemicals prior to slide-mounting). [file 13071_2020_3957_MOESM2_ESM.tif]

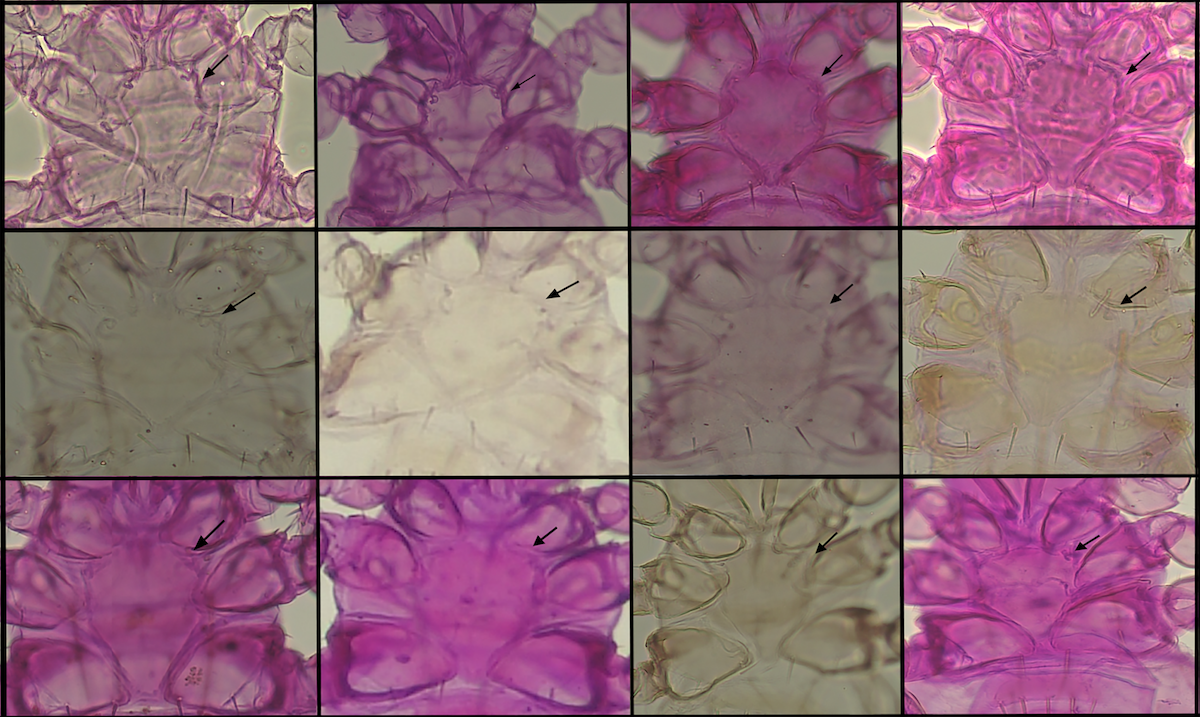

Supplement: Supplementary file 3 — Additional file 3: Figure S3. Thoracic sternal plates of Polyplax spinulosa collected from different species of Rattus: (1) ♂ from R. fuscipes; (2) ♀ from R. fuscipes; (3) ♂ from R. lutreolus; (4) ♀ from R. lutreolus; (5) ♂ from R. sordidus; (6) ♀ from R. sordidus; (7) ♂ from R. tunneyi; (8) ♀ from R. tunneyi; (9) ♂ from R. villosissimus; (10) ♀ from R. villosissimus; (11) ♂ from R. rattus; and (12) ♀ from R. rattus. [file 13071_2020_3957_MOESM3_ESM.tif]

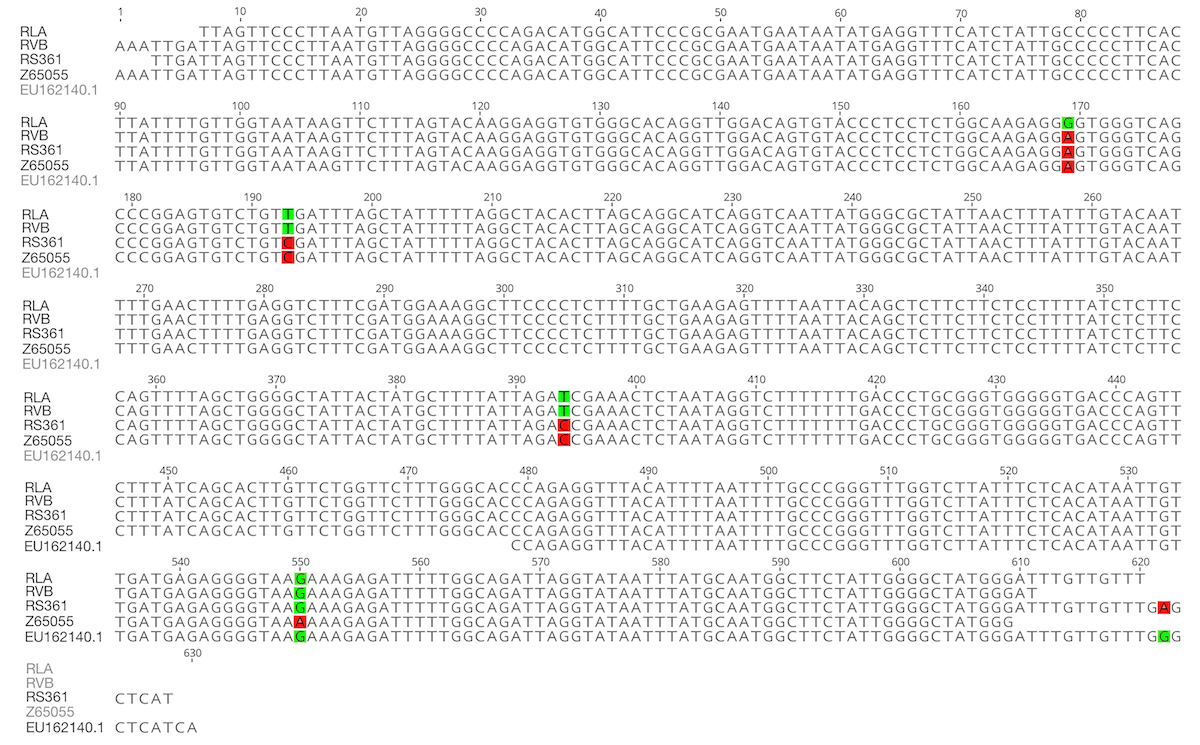

Supplement: Supplementary file 4 — Additional file 4: Figure S4. Sequence analysis of the mitochondrial cox1 gene of RLA, RVB, RS361 and Z65055 in comparison with EU162140. The four sites with nucleotide variation are indicated by red and green shading. [file 13071_2020_3957_MOESM4_ESM.tif]
